# Dominant Effects of Short-Chain Branching on the Initial Stage of Nucleation and Formation of Tie Chains for Bimodal Polyethylene as Revealed by Molecular Dynamics Simulation

**DOI:** 10.3390/polym11111840

**Published:** 2019-11-08

**Authors:** Yanling Hu, Yunqi Shao, Zhen Liu, Xuelian He, Boping Liu

**Affiliations:** 1 Shanghai Key Laboratory of Multiphase Material Chemical Engineering, East China University of Science and Technology, Shanghai 200237, China; huyl@mail.ecust.edu.cn (Y.H.); yunqi_shao@mail.ecust.edu.cn (Y.S.); liuzhen@ecust.edu.cn (Z.L.); 2 College of Materials and Energy, South China Agricultural University, Guangzhou 510642, China

**Keywords:** short-chain branching distribution, methylene sequence length, bimodal polyethylene, molecular dynamics simulation, crystal nucleation, tie chains

## Abstract

The molecular mechanism of short-chain branching (SCB), especially the effects of methylene sequence length (MSL) and short-chain branching distribution (SCBD) on the initial stage of nucleation, the crystallization process, and particularly the tie chain formation process of bimodal polyethylene (BPE), were explored using molecular dynamics simulation. This work constructed two kinds of BPE models in accordance with commercial BPE pipe resins: SCB incorporated in the long chain or in the short chains. The initial stage of nucleation was determined by the MSL of the system, as the critical MSL for a branched chain to nucleate is about 60 CH_2_. SCB incorporated in the long chain led to a delay of the initial stage of nucleation relative to the case of SCB incorporated in the short chains. The increase of branch length could accelerate the delay to nucleation. The location of short chain relative to the long chain depended on the MSL of the short chain. As the MSL of the system decreased, the crystallinity decreased, while the tie chains concentration increased. The tie chains concentration of the BPE model with branches incorporated in the long chain was higher than that with branches incorporated in the short chain.

## 1. Introduction

Polyethylene (PE) is the most widespread thermoplastic used in various consumer goods as a result of its outstanding end-use properties and high performance-price ratio. Polymer chain microstructure, such as short-chain branching distribution (SCBD) and molecular weight distribution (MWD), can dominantly influence the properties of PE. Revealing the molecular mechanism of these chain microstructures evolution will be very important for the analysis of the relationship between polymer structure and polymer properties and also the development of high-performance polymer materials. For instance, due to the well-controlled SCBD and MWD, bimodal polyethylene (BPE) shows excellent mechanical properties and good processability [1,2]. Therefore, it can be widely used for high-performance pipes and high-strength films [3,4,5,6]. Molecular dynamics (MD) simulation acts as a useful and powerful tool that can be used to investigate the molecular mechanism of chain microstructures evolution at the molecular level.

The crystallization of polymer is known as a two-step process, first, primary nucleation which can be characterized by the emergence of nanometer-sized embryos [7,8], and second, crystal growth which is the subsequent growth process of the embryos. Although the crystal growth process has been extensively investigated by experiments and simulations [9,10,11,12,13,14,15,16], the first stage, primary nucleation, did not receive too much attention [17,18]. Anwar et al. conducted MD simulations of nucleation in a polyethylene (C500) melt, and discovered that the nucleation process can be described in three stages: chain segments alignment, chain straightening and densification [19]. Zerze et al. proposed an alternative method to define ordered domains and growing nuclei based on an eicosane system [20]. Rutledge et al. calculated the interfacial free energy and nucleation rate based on a cylinder model in the homogenous nucleation of C150 and C1000 chains [21,22]. Su et al. observed that the *trans*-rich sequences aggregate in the formation process of the embryo [23]. Sanmartín et al. reported that the nucleation processes of branched ultralong n-alkanes start later than that of linear chain system [24]. In our previous work, the branch effects on the crystallization process of PE chain were explored and *trans*-rich phenomenon in the initial stage of nucleation was observed [25]. The crystallization mechanism of a series of linear PE chains with different chain length under a wide range of temperature was also explored [26]. Most recently, the molecular-level understanding of the short-chain branching (SCB) effect on the nucleation process of BPE has been revealed using bimodal blends of a short PE chain (C1000) and a long PE chain (C10000) with SCB distributed on either the short PE chain or the long PE chain [27]. BPE models with SCB incorporated in the long PE chain nucleate much later than those with SCB incorporated in the short PE chain. Moreover, the nucleation process can be further delayed when the branch length is increased. However, the BPE models built in our previous work are relatively simple, and seems to deviate from the normal industrial BPE a lot. The weight ratio of the long PE chain to the short PE chain in the BPE models is about 10:1, while the normal industrial BPE is about 1:1. Also, there is no current experimental work or simulation research about the methylene sequence length (MSL, the number of methylene units between any two adjacent branches) effects on the initial stage of the nucleation process of BPE, such as the formation of precursors, and the transformation of precursors to stable nuclei. So further study of the effects of SCB and MSL on the initial stage of nucleation for more complex BPE models, which are much closer to the normal industrial BPE, is very necessary.

Improved understanding of the branch effect on PE crystallization has been achieved due to a large number of investigations through experiments and simulations. Mandelkern et al. discovered that melting temperatures of ethylene copolymers decreased with the increasing branch content, but were independent of branch length [28,29]. Liu et al. explored branch effects on ethylene-1-hexene copolymer crystallization and found that the lamellae thickness decreased when branch content increased, accompanied by the transition of the final morphologies [30]. Wagener et al. [31,32,33,34,35,36,37,38] prepared branched polyethylene with SCB evenly incorporated in the linear backbone by using the acyclic diene metathesis (ADMET) method. They observed that melting temperatures decreased as branch content increased, and branch length began to show great impact on the melting temperatures only at a high branch content (e.g., >50 SCB/1000 backbone carbons). As it is a challenge for the current experimental techniques to observe much smaller spatial scales and time scales of physical changes, computer simulation provides another valuable vehicle to disclose the molecular mechanism of SCB on PE crystallization. Zhang et al. [39,40,41,42] performed a series of MD simulations to study the branch effects on the crystallization process of low-density polyethylene, and found that branch content and branch length could greatly influence the lamellar structure formation process. Ramos et al. [43] reported that the lamellar thickening processes of the branched polyethylene samples were impeded when compared with that of the linear polyethylene system. Choi et al. [44,45,46] investigated the branch effects on the structure of linear low-density polyethylene, and identified a critical branch content (38.5 SCB/1000 backbone carbons) where the branch length began to influence the order parameter. Rutledge et al. [47] observed that the interfacial region’s thicknesses of linear polyethylene systems were bigger than those found for branched systems. Although many research works focused on the PE crystallization, the investigation of the branch effects on BPE crystallization is still very lacking. Krishnaswamy et al. [48] examined the SCBD effect on crystallization behavior of high-density polyethylene (HDPE) using blends of low and high molecular weight PE chains with branches distributed on either the low or the high molecular weight PE chain. In our previous study, the placement of branches on the long PE chain resulted in much lower crystallinity as compared with that of branches on the short PE chain when branch content exceeded 5 SCB/1000 backbone carbons [27]. This critical branch content was not influenced by branch length. BPE models with branches incorporated in the long chain tended to form more tie chains relative to those with branches incorporated in the short chain. The tie chains concentration was independent of branch length. As mentioned above, these BPE models are relatively simple, deviating far from the normal industrial BPE. Moreover, the MSL effects on the crystallization process and also the tie chain formation process of BPE models were not considered as the MSL between the two different types of BPE models built in the previous work is quite different. What about if the MSL between the two kinds of BPE models is very comparable? So this is another key issue to be addressed in the present work.

The purpose of this work is to study the molecular mechanism of SCB, especially MSL, as well as the SCBD mechanism on the crystallization process of BPE. This study is an extension of our previous simulation for the simple BPE models [27]. The motivation for this study is three-fold. First, increase the number of short chain in the BPE models built in our previous study, so that these models can be much closer to the normal industrial BPE. Second, consider the MSL effect on the initial stage of nucleation of BPE. Third, investigate the MSL effects on the crystallization process and tie chain formation process of BPE. So MD simulations were carried out for two types of more complex BPE models which have the same branch content, branch length and comparable MSL, but different SCBD. The molecular mechanism of SCB and MSL on the initial stage of nucleation, also the crystal structures and especially the tie chains of BPE models were systematically studied.

## 2. Models and Methods

**Figure 1:**
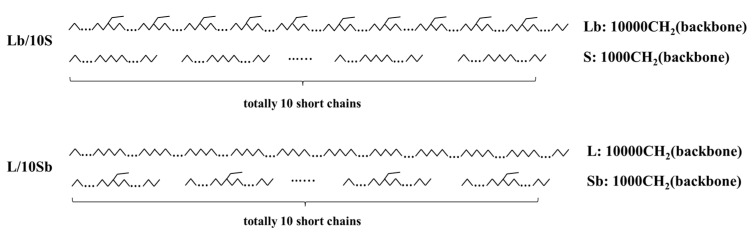
Chain architectures of two kinds of bimodal polyethylene (BPE) models (Lb/10S and L/10Sb) built in this work. Both these two kinds of BPE models consisted of one long chain (backbone: 10,000 CH_2_) and ten short chains (backbone of each short chain: 1000 CH_2_), but with different short-chain branching distribution (SCBD). One type contained branches which incorporated equally in the long chain (Lb/10S) while the other type contained branches incorporated equally in the ten short chains (L/10Sb).

As shown in Figure 1, two kinds of precisely branched BPE models were built in accordance with industrial BPE pipe resins in this study. Each kind of BPE model contained 11 chains: one long chain with 10,000 CH_2_ in backbone and ten short chains with 1000 CH_2_ in backbone for each short chain. One kind contained branches incorporated evenly in the long chain (Lb/10S), but the other kind contained branches incorporated evenly in the ten short chains (L/10Sb). It is worth noting that Lb/10S and L/10Sb systems have the same branch content, branch length but different SCBD: branches all incorporated in the long chain (Lb/10S) or in the short chains (L/10Sb). In our previous work, two kinds of simple BPE models which consist of only one long chain with 10,000 CH_2_ in backbone and one short chain with 1000 CH_2_ in backbone were built [27]. These two types of simple BPE models also have the same branch content, branch length but different SCBD: branches incorporated in the long chain (Lb/1S) or in the short chain (L/1Sb). For convenience, here we call the models built in our previous work as simple BPE models (Lb/1S and L/1Sb), and models built in the present work as complex BPE models (Lb/10S and L/10Sb). Unless otherwise stated, BPE models include the simple BPE models and the complex BPE models. It is very clear that the number of short chains in the complex BPE model was ten, which was greater than that in the simple BPE model. The complex BPE model which contained about 20,000 CH_2_ in total was much larger than the simple BPE model as it contained only 11,000 CH_2_ in total. Also, the molecular weight distribution of the complex BPE model was different from that of the simple BPE model. Table 1 summarizes the details of the complex BPE models used in this work. As the branch content of normal industrial BPE is very low, which ranged from 2 to 5 branches per 1000 carbons, such as the average branch content of Borstar BPE (PE100) is about 2.1 branches per 1000 carbons [6], so in this work, we choose to study the complex BPE models with branch content ranged from 0 to 8 SCB/1000 backbone carbons. The naming method for these complex BPE models recognizes the composition of the models, also the branch type, and the branch content. For example, Lb/10S-C2-1 represents the blend of one branched long chain (Lb) with ethyl branches (C2) and ten linear short chains (10S), and the average branch content of this model is 1 SCB/1000 backbone carbons (“1”). However, L/10Sb-C2-1 represents the blend of one linear long chain (L) and ten branched short chains (10Sb) with ethyl branches (C2), and the average branch content of this model is 1 SCB/1000 backbone carbons (“1”). We also considered the branch length effect in this study. The butyl or hexyl branched complex BPE models (not shown here) were designed similarly to the ethyl branched complex BPE models, and the differences are the branch length. Each methylene or methyl unit, CH_2_ or CH_3_ unit, in all the models built in this work was treated as a united atom in order to simplify the calculation [49].

MD simulations were carried out using GROMACS 4.5.5 [50], with the Dreiding II force field [51]. The Dreiding II force field is a generic force field that is very widely used for PE due to the hexagonal crystal structure of PE chains that can be obtained in simulations [23,27,52,53,54,55]. Due to the lower torsional barrier heights from the *gauche* conformation to the *trans* conformation, polymer crystallization can be faster, which will lead to a significant saving in CPU time. Choi et al. discussed the effects of this force field on the folding process of PE chains and found that the choice of force fields was very important for PEs with high crystallinity [56]. Reduced units for time, distance, temperature and energy were used in this work: (1) The normalized time (t*) was given in τ units, and we set t* = 1 τ which stands for the real-time of 1 ps. (2) The normalized distance (l*) was given in σ units, and we set l* = 1 σ which stands for the real distance of 1 nm. (3) The normalized temperature (T*) was calculated as T* = k_B_T/ε, where T is the real temperature, k_B_ is the Boltzmann constant (k_B_ = 8.3145 × 10^−3^ kJ mol^−1^ K^−1^), and ε is the van der Waals interaction parameter (ε = 0.8301 kJ mol^−1^) in a 12–6 Lennard–Jones potential of Dreiding II force field [51]. We set the normalized temperature T* = 8.0, which stands for the real temperature T = 800 K. (4) The normalized energy (E*) was given in ξ units, and we set E* = 1 ξ which stands for the real energy of 1 kJ/mol. The velocity Verlet algorithm was chosen for the integration of the equations of motion, where the time step was set at 2 × 10^−3^ τ.

A canonical NVT ensemble was used for a vacuum state research, and the Nose–Hoover thermostat with a time constant of 0.5 τ was used for the control of the temperature. In the simulation, the polymer was placed in the center of a cubic box, and the normalized distance from the end of the polymer chain to the closest edge of the box was set at about *l*_e_* = 25 σ (the corresponding real distance is about 25 nm, and the box size effect was discussed in the Supplementary Material). So the box size was fixed as ′very large′ to contain the polymer so that enough empty space is reserved to maintain a vacuum condition where there was no boundary condition for all the polymer chains [26,42,45,52,53]. The density of the cubic box was kept constant throughout the crystallization process. However, the density change of the BPE models during the crystallization could be reflected through the variation of order parameters of the polymer chains which will be discussed in the following section. The calculations of all reported static properties of the polymer chains (e.g., order parameters) center on the coordinate of every CH_2_ unit and these surrounding voids are ignored. 

For the sake of achieving an equilibrium amorphous state at the first opportunity, the temperature adopted in this study was higher than that observed in the real experiment, and the temperature here was modified to the normalized temperature T* (see Supplementary Material) [26,52]. The complex BPE models with an all-*trans* conformation were first minimized to remove high-energy overlaps, and then were annealed at T* = 8.0 for t* = 3.0 × 10^4^ τ to obtain equilibrium amorphous coils. Here T* = 8.0 was corresponding to the real experimental temperature which was higher than the melting temperature of BPE, a detailed explanation can be found in the supplementary material. Then the amorphous structure was quenched from T* = 8.0−3.0 with a cooling rate of Γ* = 2.5 ×10^−4^ τ^−1^, and at last the simulations were performed at T* = 3.0 for another t*= 4 × 10^3^ τ. 

In order to quantify the crystallinity of the polymer chains, we followed a site order parameter (SOP) method proposed by Yang et al. [52]. The SOP of site k is calculated as follows, where i and j are any two-unit vectors in the domain with radius *R* = 0.55σ surrounding the site:

(1) $${\mathrm{SOP}}_{k}=\frac{\langle {3\cos}^{2}\left(\varphi \right)-1\rangle }{2}=\frac{3}{2}{\langle {\left(\vec{e_{i}}\cdot \vec{e_{j}}\right)}^{2}\rangle }_{R}-\frac{1}{2}.$$

For a system which contains N sites, the SOP of this system is defined as the average of SOP_k_ of all the sites in the system:

(2) $$\mathrm{SOP}=\frac{1}{N}\sum_{k=1}^{N}{\mathrm{SOP}}_{k}.$$

The Crystallinity (X_c_) of this polymer system can then be calculated by the proportion of sites with SOP_k_ higher than 0.7 [52].

The orientation vectors in the ordered domains where SOP_k_ are higher than 0.7 are deemed to locate in the same nucleus only when the distance between any two of the orientation vectors is less than about 0.68 σ, and also the average cosine of angle between these vectors is higher than 0.82 [20]. So, the entire ordered region is grouped into different clusters, and clusters that contain more than 30 monomers are considered as nuclei or crystal domains.

In addition to the site order parameter, the dihedral distribution of the polymer system was also calculated. The proportion of a dihedral angle m along the backbone of the polymer chains is defined as follows:

(3) $$P_{m}=\frac{N_{m}}{\sum N_{m}},$$

where *N*_m_ is the number of atoms with the dihedral angle m (m = 0° − 360°). The *trans* state corresponds to the torsion angle between 180° ± 15°.

To determine the entanglement density of this polymer system, a parameter named as interchain contact fraction (ICF) was introduced by Yang et al. [52]. It can be calculated using the equation below:

(4) $$\mathrm{ICF}=\frac{\sum N_{\mathrm{inter}}{(r}_{n})}{\sum N_{\mathrm{total}}{(r}_{n})},$$

where *N*_inter_(*r*_n_) signifies the number of interchain units around a certain unit at distance *r*_n_, and *N*_total_(*r*_n_) signifies the number of total units (*N*_total_(*r*_n_) = *N*_inter_(*r*_n_) + *N*_intra_(*r*_n_)) around the same unit. The distance r_n_ ranges from 0.48 to 0.52 σ.

## 3. Results and Discussion

### 3.1. Induction Time of Precursor Formation

**Figure 2:**
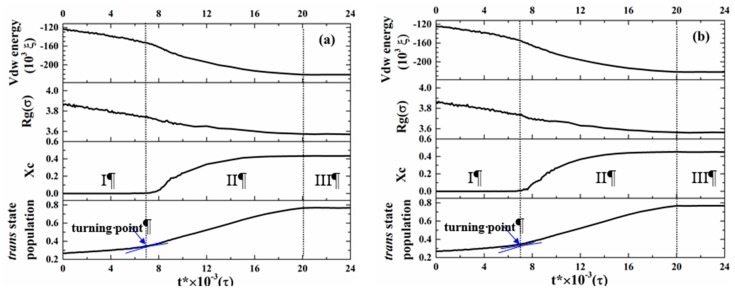
Evolution curves of van der Waals energy, R_g_, X_c_ and *trans*-state population for (**a**) L/10Sb-C4-1 model and (**b**) Lb/10S-C4-1 model. Note that these parameters of the model are the averages of the long chains and short chains in the model. The crystallization process of complex BPE is divided into three stages: I: an induction period, II: a rapid growth stage, III: a final stable period.

We first concentrated our attention on the precursor formation during the induction period of BPE models. The time evolutions of non-bonding potential energy (vdW energy) and structure parameters (R_g_, X_c_ and *trans*-state population) for L/10Sb-C4-1 model and Lb/10S-C4-1 model are shown in Figure 2, respectively. There are three stages according to the evolutions of these parameters for the two models: an induction period, a rapid growth stage and a final stable period. For instance, vdW energy decreased slightly at the first t* = 7 × 10^3^ τ, then it decreased remarkably until t* = 2.0 × 10^4^ τ, and after 2.0 × 10^4^ τ, it remained almost unchanged. The similar phenomena were also observed for the corresponding Rg, X_c_ and *trans*-state population of the two models. During the induction period, R_g_ decreased slightly, and *trans*-state population increased slowly, while X_c_ kept at around 0. A new *trans*-rich structure (also precursor) formed in this period due to the transformation of dihedral angle of bonds from random *trans*/*gauche* states to *trans* states [25]. However, the increase of crystallinity was found to lag behind this *trans*-rich phenomenon. There was a turning point from the induction period to the rapid growth stage, which was the endpoint of the induction period, as well as the induction time of precursor formation. Before this turning point, X_c_ was stable at around 0, and after this turning point, X_c_ began to increase. Therefore, we define this turning point as the endpoint of the induction period where the crystallinity of the precursor reached about 0.005 [25].

**Figure 3:**
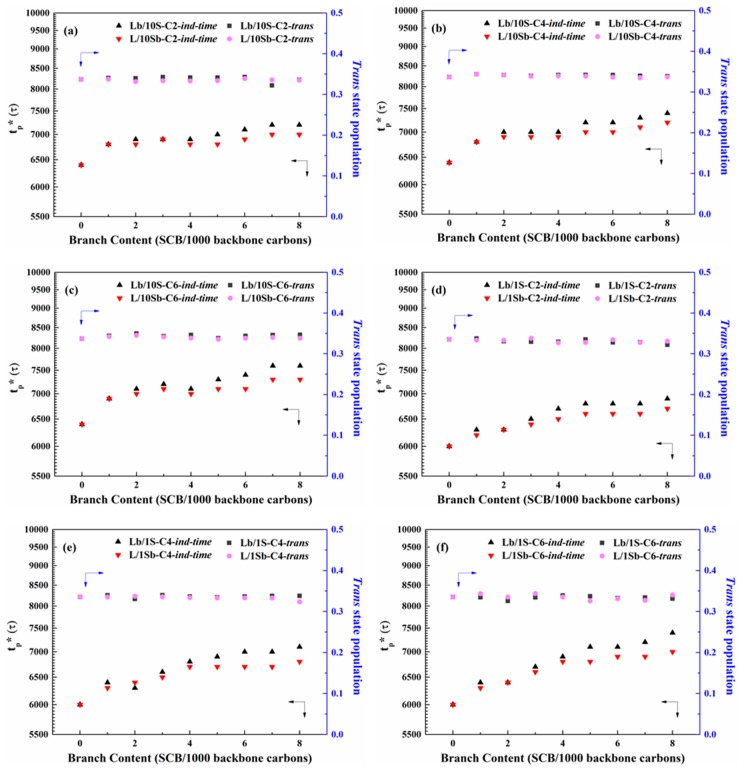
SCBD dependence of induction time (*t*_p_*) and *trans*-state population for the two kinds of complex BPE models with (**a**) ethyl branches, (**b**) butyl branches and (**c**) hexyl branches and the two kinds of simple BPE models with (**d**) ethyl branches, (**e**) butyl branches and (**f**) hexyl branches.

The corresponding *trans* state (180° ± 15°) populations along the backbone of all the two types of precisely branched BPE models at the induction time are about 0.33, as shown in Figure 3. The SCBD dependence of induction time (*t*_p_*) and the corresponding *trans*-state population for the two kinds of complex BPE models with different branch length [Figure 3a–c] and the two types of simple BPE models with different branch length [Figure 3d–f] are clearly shown in Figure 3, respectively. For the case of ethyl branches incorporated in the ten short chains, we use L/10Sb-C2-*ind-time* and L/10Sb-C2-*trans* to represent the induction time and the corresponding *trans*-state population of this system, but for the case of ethyl branches incorporated in the long chain, we use Lb/10S-C2-*ind-time* and Lb/10S-C2-*trans* instead. The naming conventions for those butyl or hexyl branched BPE models are similar to the ethyl branched systems. 

Comparing the ethyl branched Lb/10S system and Lb/1S system, the induction time of the Lb/10S-C2 system was longer than that of Lb/1S-C2 system. When the branch length changed to butyl and hexyl, the results were also very similar. This may be explained by the lower MSL of Lb/10S system when compared with that of Lb/1S system, as shown in Table S1 in the supplementary material. The details of the MSL of the simple BPE models and the complex BPE models are shown in Table S1 (in the supplementary material). Hence, it seems that the induction time of precursor formation is determined by the MSL of the system. The induction time of the precursor formation prolonged with the decrease of the MSL of the system.

The induction time increased with the increasing branch content for all the complex BPE models (L/10Sb system and Lb/10S system). This behavior is consistent with that of a single branched PE chain [25]. For the two kinds of complex BPE models, it is clearly observed that the induction time of Lb/10S systems is longer than that of L/10Sb systems. More interesting, the difference of induction time of the precursor formation between these two kinds of complex BPE models increased when branch length increased from the ethyl branch to hexyl branch. The similar phenomena were observed for the simple BPE models, as shown in Figure 3d–f. Therefore, for all the two kinds of BPE models, SCB incorporated in the long chain leads to a delay of induction period relative to the case of SCB incorporated in the short chain, and this delay effect increased with the increasing branch length.

**Figure 4:**
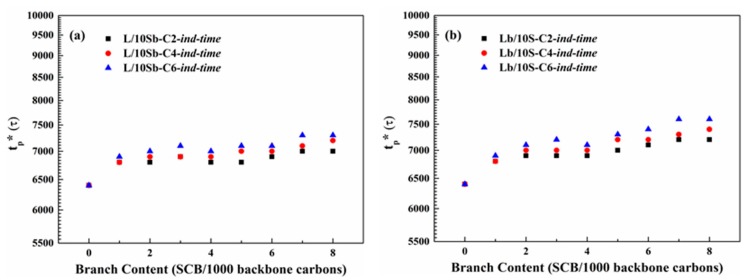
Branch length dependence of induction time (*t*_p_*) for (**a**) L/10Sb systems and (**b**) Lb/10S systems.

Figure 4 depicts the branch length dependence of induction time for the two kinds of complex BPE models. As observed, the induction time increased as branch length increased for both L/10Sb systems and Lb/10S systems, but Lb/10S systems show a bigger difference. For the simple BPE models, the phenomena were also very similar, as shown in Figure S2 in the supplementary material. Hence, the induction periods for all the BPE models were delayed as branch length increased. Additionally, this delay effect will become worse for BPE models with SCB incorporated in the long chain as compared to that with SCB incorporated in the short chain.

### 3.2. The Transformation of Precursors to Stable Nuclei

Parts of the precursors forming during the induction period will be transformed into stable nuclei. The first stable nucleus means the first nucleus transformed by the precursors, meanwhile, this nucleus could exist for a relatively long period (often several hundred τ) [27]. Note that if the formed first nucleus disappears very soon (less than 100 τ), it was not considered as the first stable nucleus. The formation time of the first stable nucleus was defined as the time when this nucleus emerged. We then traced the first stable nucleus formation process and attained the details of the nucleus structure. In order to analyze the nucleus structure, we introduced another parameter named long-chain proportion (LCP) [27]. LCP corresponds to the ratio of long-chain carbons to the total carbons in the first stable nucleus. 

**Figure 5:**
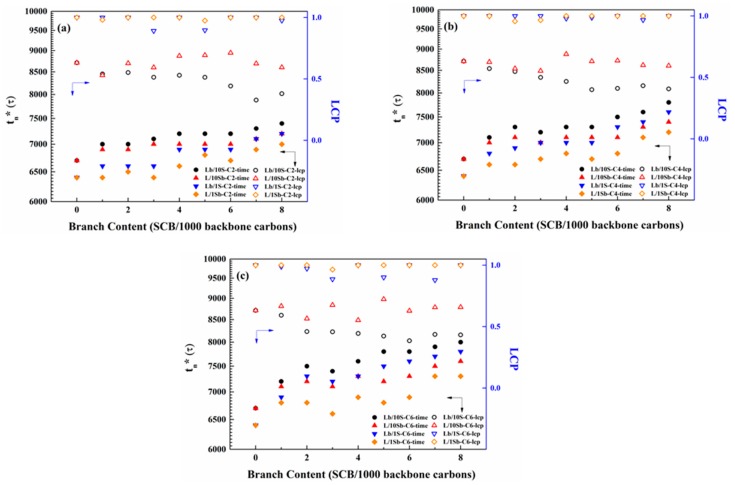
SCBD effect on the first stable nucleus formation time (*t*_n_*) and long-chain proportion (LCP) for precisely branched complex BPE models and simple BPE models with (**a**) ethyl branches, (**b**) butyl branches and (**c**) hexyl branches. LCP: the ratio of long-chain carbons to the total carbons in the first stable nucleus [27]. Reprinted the data for the simple BPE models with permission from Ref. [27]. Copyright 2018 Elsevier Ltd.

Figure 5 exemplifies the SCBD effect on the first stable nucleus formation time (*t*_n_*) and the corresponding LCP for the BPE models with different branch lengths. Comparing the Lb/10S-C2 system with the Lb/1S-C2 system, the first stable nucleus formation time of the Lb/10S-C2 system was longer than that of the Lb/1S-C2 system. It was also observed that the differences of the first stable nucleus formation time between these two systems decreased with the increasing branch content. Similar phenomena were also observed for the butyl- or hexyl-branched Lb/10S systems and Lb/1S systems. This may also result from the lower MSL of the Lb/10S system when compared with that of the Lb/1S system, and much smaller MSL differences when the branch content increased, as shown in Table S1 in the supplementary material. Therefore, the first stable nucleus formation time was influenced by the MSL of the system. The first stable nucleus formation time increased with the decrease of MSL of the system.

It is observed that for the two different kinds of complex BPE models, the first stable nucleus formation time both increase with the increasing branch content. More interesting, the first stable nucleus formation time of L/10Sb systems were much shorter than that of Lb/10S systems. For the simple BPE models, the first stable nucleus formation time of L/1Sb systems was also much shorter than that of Lb/1S systems [27]. Hence, SCB incorporated in the long chain will result in a delay to the nucleation process when compared with that of SCB incorporated in the short chain.

As depicted in Figure 5a, the LCP of Lb/10S-C2 system in the nucleus was much lower than that of the Lb/1S-C2 system. Also, the LCP of the L/10Sb-C2 system was lower than that of the L/1Sb-C2 system. When the branch length increased to butyl or hexyl, the phenomena were also very similar, as shown in Figure 5b,c. This may be attributed to the different MSL between the simple BPE model and the complex BPE model. As the MSLs of Lb/10S systems are much shorter than those of Lb/1S systems (see Table S1 in supplementary material), it was more difficult for the long-chain components of Lb/10S systems to nucleate in comparison with those of Lb/1S systems, thereby leading to much lower LCPs of Lb/10S systems. Due to the longer MSLs of L/10Sb systems in comparison with those of L/1Sb systems (see Table S1 in the supplementary material), short-chain components in L/10Sb systems can participate in the nucleation process, while those in L/1Sb systems cannot. Therefore, the LCPs of L/10Sb systems will be much lower than those of L/1Sb systems. Moreover, it is intriguing to find that the MSLs of L/10Sb systems are all longer than about 60 CH_2_, and the short chains in L/10Sb systems will participate in the nucleation process. However, the MSLs of L/1Sb systems are almost shorter than 60 CH_2_, and the long chains in L/1Sb systems will be predominant in the nucleation process. Hence, it seems that only when the MSLs of the BPE models are longer than 60 CH_2_, then the branched chains can nucleate. Ungar et al. reported that the shortest folded chain length was 150 carbons when polymer crystallized from solution [57]. Zerze et al. [20] and Rutledge et al. [22] found such a critical fold length was 20–30 carbons when polyethylene crystallized from the melt. Flory discovered that at each crystallization temperature there was an MSL for a polymer chain to crystallize [58]. The critical MSL (60 CH_2_) for a branched chain to nucleate obtained in this work was longer than the critical fold length (20–30 carbons) reported about polymer crystallization from the melt. But it was shorter than the critical fold length (150 carbons) reported about the polymer crystallization from solution, which is due to the interaction between polymers and solvent molecules in solution. So in general, the critical MSL (60 CH_2_) for a branched chain to nucleate derived from this work is reasonable.

Obviously, the LCPs of L/10Sb systems in the nucleus increased slightly as branch content increased. However, the LCPs of Lb/10S systems decreased with the increasing branch content, and all were lower than 60%. This may be due to the decrease of MSLs of the long chains in Lb/10S systems and the short chains in L/10Sb systems, respectively. More interesting, the LCPs of L/10Sb systems are higher than those of Lb/10S systems, which suggest that when the MSLs of them are comparable (see the supplementary material Table S1), both short chain and long chain of them can take part in the nucleation process, and SCB incorporated in the long chain will hinder the long chain from nucleating relative to the case of SCB incorporated in the short chains. Therefore, the nucleation processes of BPE models were determined by the MSLs of them. The critical MSL for a branched chain to nucleate is about 60 CH_2_. The percentage of the long-chain carbons to nucleate increased as the MSL of the model increased. It needs to mention that the LCPs of L/1Sb systems and Lb/1S systems are all higher than 90% although the MSLs of them decreased with the increasing branch content. It may be explained by the strong molecular weight effect of the long chain to the short chain on the chain conformational relaxation time. As a result, long chain predominates in the nucleation for the simple BPE models. But for the complex BPE models, it seems that MSL has a greater influence than molecular weight, thereby short chains and long chains of the models will nucleate together to form the first stable nucleus. 

**Figure 6:**
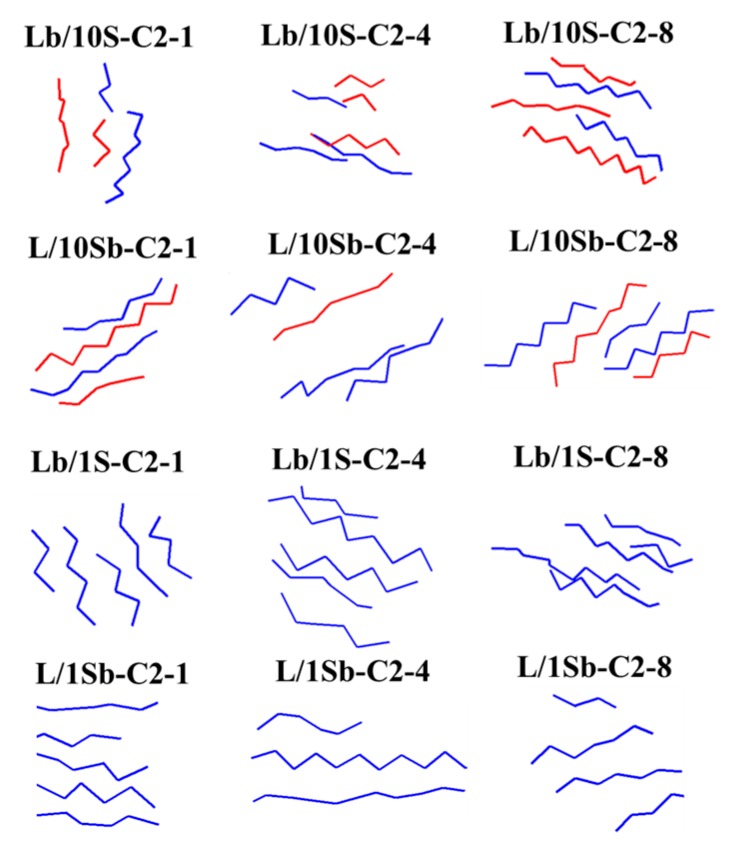
Microstructures of the first stable nucleus formed in the branched complex BPE models and simple BPE models with ethyl branches. Blue monomers represent the long-chain carbons in the first stable nucleus and red monomers represent the short-chain carbons in the first stable nucleus.

To better understand the formation process of the first stable nucleus, we analyzed the microstructures of the first stable nucleus formed in the ethyl branched complex BPE models and simple BPE models, as shown in Figure 6. The long-chain carbons in the first stable nucleus are colored in blue, and the short-chain carbons are colored in red. It is observed that long chains and short chains all participate in the formation processes of the first stable nucleus for the complex BPE models (Lb/10S-C2 system and L/10Sb-C2 system), while for the simple BPE models (Lb/1S-C2 system and L/1Sb-C2 system), only long chains take part in the formation processes of the first stable nucleus. So the LCP of Lb/10S-C2 system was much lower than that of Lb/1S-C2 system. Similar behavior was also found for the L/10Sb-C2 system and L/1Sb-C2 system. The different nucleus structure between the complex BPE model and the simple BPE model was mainly explained by the different MSL between these models. As the MSL of the Lb/10S-C2 system is shorter than that of the Lb/1S-C2 system, the short chains in the Lb/10S-C2 system can get the chance to take part in the nucleation. Also, the MSL of the L/10Sb-C2 system is much longer than that of the L/1Sb-C2 system, so the short chains in the L/10Sb-C2 system can obtain the opportunity to participate in the nucleation. We also observed that when the MSL of the polymer chain is longer than 60 CH_2_, increasing the proportion of the short chain in the model can increase the probability of participating in the nucleation of the short chain. Moreover, the percentage of long-chain carbons in the first stable nucleus of the L/10Sb-C2 system was higher than that of short chains, and increased a little with increasing branch content. But for the Lb/10S-C2 system, the percentage of long-chain carbons in the first stable nucleus was much lower than that of short chains at high branch content (>4 SCB/1000C), and apparently decreased with the increasing branch content. The LCP of L/10Sb-C2 system was higher than that of Lb/10S-C2 system. These observations are in agreement with the LCPs of ethyl branched complex BPE models and ethyl branched simple BPE models, as shown in Figure 5. The similar phenomena are also observed for the butyl or hexyl branched systems.

**Figure 7:**
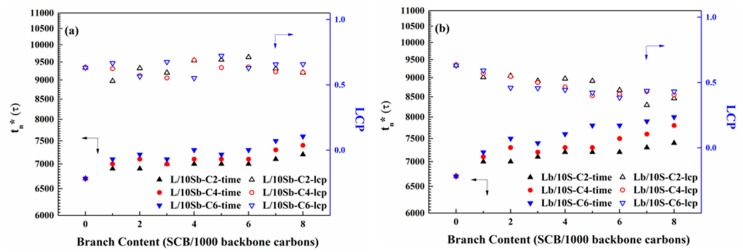
Branch length dependence of the first stable nucleus formation time (t_n_*) and LCP for (**a**) L/10Sb systems and (**b**) Lb/10S systems.

The branch length dependence of the first stable nucleus formation time and LCP for the two kinds of complex BPE models are illustrated in Figure 7. The first stable nucleus formation time increased with the increasing branch length for both L/10Sb systems and Lb/10S systems, and the increase of Lb/10S systems was much more significant. This observation is also found in the simple BPE models [27]. Therefore, the nucleation processes for the two kinds of BPE models are delayed when the branch length increases. Additionally, the delay effect of branch length on the nucleation will become much worse when branches were incorporated in the long chain as compared to the case of branches incorporated in the short chain. A similar phenomenon was also observed for the ultralong n-alkanes systems that the nucleation is delayed when the branch length increases [24]. The LCPs of L/10Sb systems with a different branch length are in a range of 50%–70%, which are insensitive to branch length, as depicted in Figure 7a. For Lb/10S systems, the LCPs are in a range of 35%–60%, which are also independent of branch length, as depicted in Figure 7b. Hence, the LCPs of L/10Sb systems and Lb/10S systems are not influenced by branch length. For the two kinds of simple BPE models, the LCPs are also independent of branch length [27].

### 3.3. Crystal Structures

In order to deduce how SCB affects the chain crystal structures of the BPE models, we have computed the order parameter and crystallinity of the systems. Crystallization curves of different branch content for the two kinds of complex BPE models with different branch length, including ethyl, butyl and hexyl branches are shown in Figure S3 in the supplementary material. For the two kinds of ethyl branched complex BPE models, the values of X_c_ all decreased with the increasing branch content. But the values of X_c_ of the Lb/10S-C2 systems decreased more obviously with the increasing branch content than that of L/10Sb-C2 systems, as shown in Figure S3a,b (in supplementary material). When branch length changed to butyl or hexyl, the phenomena were also very similar (see Figure S3c–f in supplementary material) These observations are consistent with that of simple BPE models [27].

**Figure 8:**
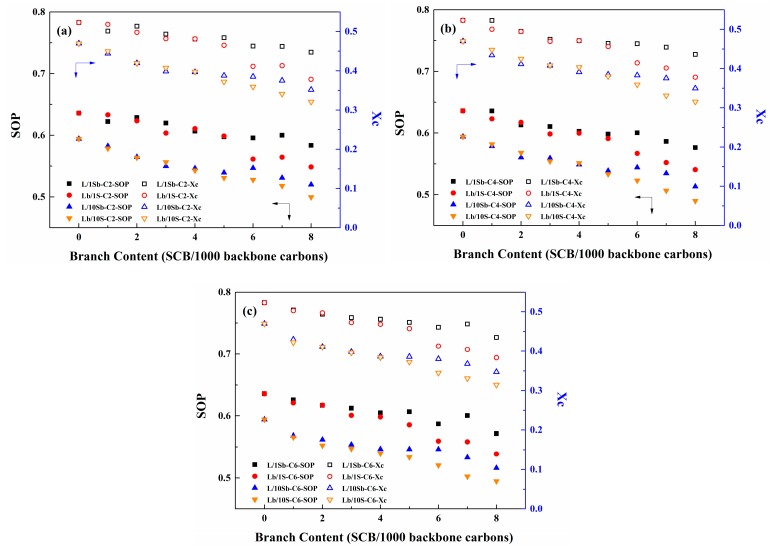
SCBD effect on the X_c_ and site order parameter (SOP) of the complex BPE models and simple BPE models with (**a**) ethyl branches, (**b**) butyl branches and (**c**) hexyl branches. Reprinted the data for the simple BPE models with permission from Ref. [27]. Copyright 2018 Elsevier Ltd.

Figure 8 shows the SCBD effect on the Xc and SOP of the complex BPE models and the simple BPE models with different branch lengths. Comparing the Lb/10S-C2 system and the Lb/1S-C2 system, the Xc and SOP of the Lb/10S-C2 system were lower than that of the Lb/1S-C2 system, as shown in Figure 8a. When the branch length increased to butyl or hexyl, similar phenomena were also observed, as depicted in Figure 8b,c. This may be also due to the lower MSL of the Lb/10S system when compared with that of the Lb/1S system (see the Supplementary Material Table S1). Hence, the crystallinity of the polymer is influenced by the MSL of the system. The crystallinity of polymer reduced with the decrease of MSL of the system. It needs to be pointed out that the Xc and SOP of the L/10Sb system are also lower than that of the L/1Sb system despite the MSL of the L/10Sb system being higher than that of the L/1Sb system. This may be attributed to the fact that more nuclei will form in the L/10Sb system when compared with the L/1Sb system, as shown in Figure S4 in the supplementary material (only shows the nuclei number of ethyl branched systems, the results of systems with butyl or hexyl branches are very similar), which may lead much lower crystallinity of the L/10Sb system.

As observed in Figure 8a, the X_c_ and SOP of the ethyl branched complex BPE models (L/10Sb system and Lb/10S system) are not influenced by the two different SCBD up to a branch content of about 5 SCB/1000C. Above 5 SCB/1000C, X_c_ and SOP of L/10Sb systems are much larger than that of Lb/10S systems. The first observation coincides with the findings of Krishnaswamy et al. where a similar density for two kinds of HDPE blends that have matched MWD and average branch content (3 SCB/1000C) were observed [48]. Hence, it seems that the SCBD effect becomes remarkable only at a high branch content (>5 SCB/1000C). Moreover, this critical branch content does not change when the branch length increases to butyl or hexyl, as shown in Figure 8b,c. Also, these observations accord well with that of the simple BPE models [27]. More interestingly, the differences in X_c_ (also SOP) between Lb/10S systems and L/10Sb systems were much lower than those between Lb/1S systems and L/1Sb systems when branch content exceeds 5 SCB/1000C. This may result from the lower MSL differences between the two types of complex BPE models when compared with the two types of simple BPE models. Therefore, the SCBD effect on crystallinity of the complex BPE models was much weaker than on that of the simple BPE models. Nevertheless, the critical branch content is independent of branch length, also the different MSL differences.

The branch length dependence of X_c_ and SOP for the two kinds of complex BPE models are depicted in Figure S5 in the supplementary material. Obviously, the X_c_ and SOP of both the two kinds of complex BPE models are insensitive to branch length. In our previous works, the X_c_ and SOP of simple BPE models and single PE model chain are also independent of branch length [25,27]. Wagener and co-workers discovered that the crystallinity of PE with branches evenly incorporated in each 21st carbon and 39th carbon were insensitive to branch length [34,37]. The effects of branch length on the crystallization kinetics of some ethylene copolymers were also invisible [29,59,60]. Choi et al. also reported that the order parameter of precisely branched polyethylene was not influenced by branch length at low branch content (<30 SCB/1000C) [46]. Hence, the crystallization kinetics of BPE models, including the complex BPE models and the simple BPE models, are independent of branch length.

**Figure 9:**
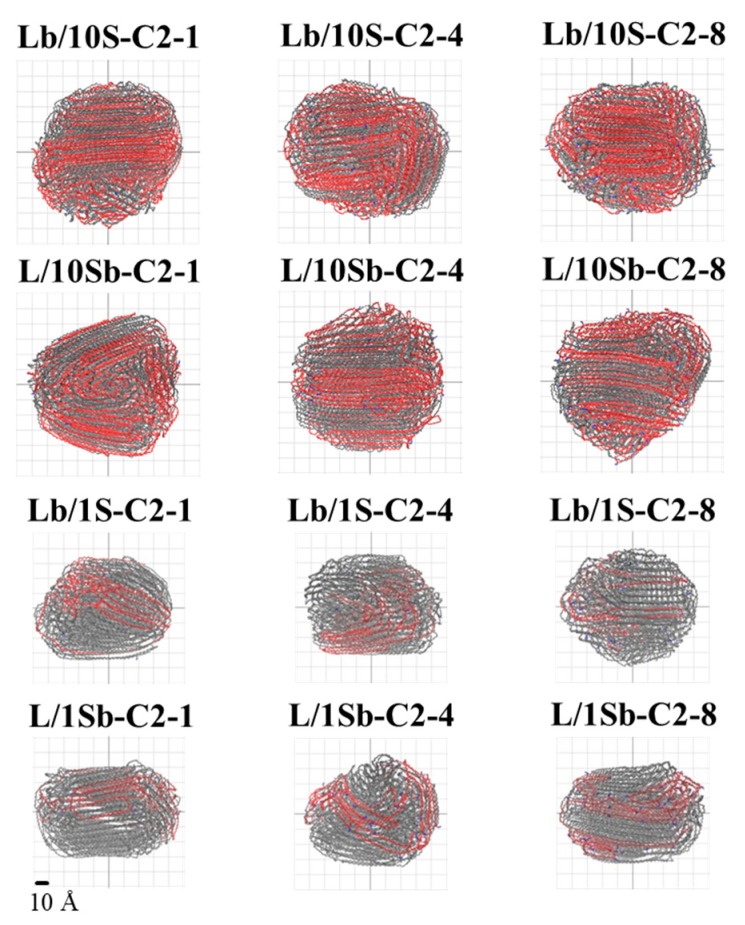
Final morphologies of ethyl branched BPE models. Final morphologies of all models correspond to morphologies of the end of the simulation. Gray monomers represent the long chain, blue monomers represent the branches, and red monomers represent the short chains. Reprinted the final morphologies of the simple BPE models with permission from Ref. [27]. Copyright 2018 Elsevier Ltd.

Figure 9 shows the final morphologies of ethyl branched complex and simple BPE models with different branch content. The final morphologies of all the BPE models correspond to the morphologies of the end of the simulation. Gray monomers represent the long chain, blue monomers represent the branches and the other colors monomers represent the short chains. As depicted in Figure 9, for the ethyl branched complex BPE models, the short chains are almost all trapped inside the long chain, no matter when the SCB incorporated in the long chain or in the short chains. For the butyl or hexyl branched complex BPE models, similar behaviors are also observed. This observation is verified by the calculated ICF difference between the initial morphology and the final morphology. The initial morphology of every model corresponds to the morphology at high temperature (T* = 8.0) during the annealing process. However, this observation is very different from that of simple BPE models: the short chain lies outside the long chain when branches incorporated in the short chain (L/1Sb system), but the short chain is trapped inside the long chain when branches incorporated in the long chain (Lb/1S system) [27]. So it seems that when the MSL of the short chain is longer than about 60 CH_2_, the short chain will be more likely to be trapped inside the long chain, as the L/10Sb system shows. But when the MSL of the short chain is shorter than about 60 CH_2_, the short chain tends to lie outside the long chain, as the L/1Sb system shows. When branches incorporated in the long chain, the short chain will tend to be trapped inside the long chain, as the Lb/10S system and the Lb/1S system show. Therefore, for the BPE models, including the simple BPE models and complex BPE models, the location of the short chain to the long chain depends on the MSL of the short chain.

**Figure 10:**
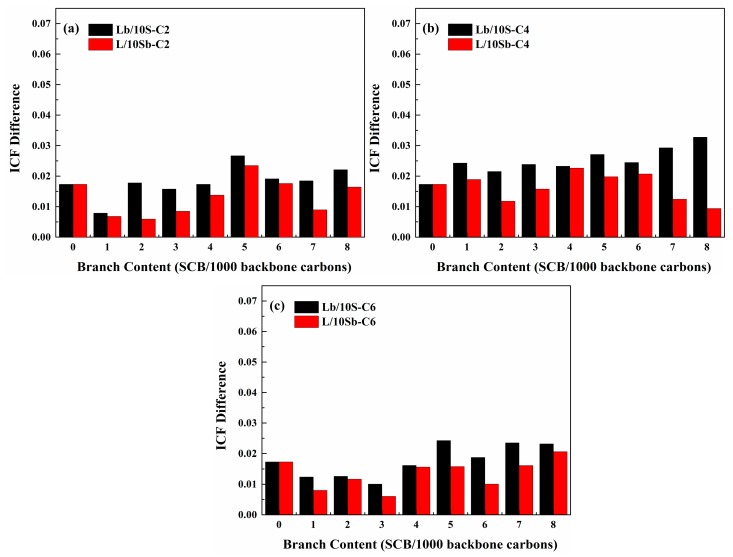
SCBD effect on the interchain contact fraction (ICF) difference (between the initial morphology and the final morphology) for the complex BPE models with (**a**) ethyl branches, (**b**) butyl branches and (**c**) hexyl branches.

The SCBD effect on the ICF differences between the initial and the final morphologies for all the complex BPE models are illustrated in Figure 10. It is clearly observed that ICF differences are positive for all the complex BPE models whenever the SCB incorporated in the long chain or in the short chains. Hence, the data indicates that after crystallization, short chains tend to get closer to the long chain, as Figure 8 shows that short chains are almost all trapped inside the long chain, so that the degree of entanglement of the models increase. This behavior is also different from that of simple BPE models: for the L/1Sb systems, ICF differences are all negative, while for the Lb/1S systems, ICF differences are positive [27]. It may also be attributed to the different MSLs of short chains between the L/10Sb systems and L/1Sb systems. For L/10Sb systems, as the MSLs of short chains are longer than 60 CH_2_, short chains tend to be trapped inside the long chain, which then caused the increase of the degree of entanglement of the L/10Sb systems. But for L/1Sb systems, when the MSLs of short chain are shorter than 60 CH_2_, the short chain will tend to lie outside the long chain, thereby the degree of entanglement of the systems will decrease.

### 3.4. Tie Chains

**Figure 11:**
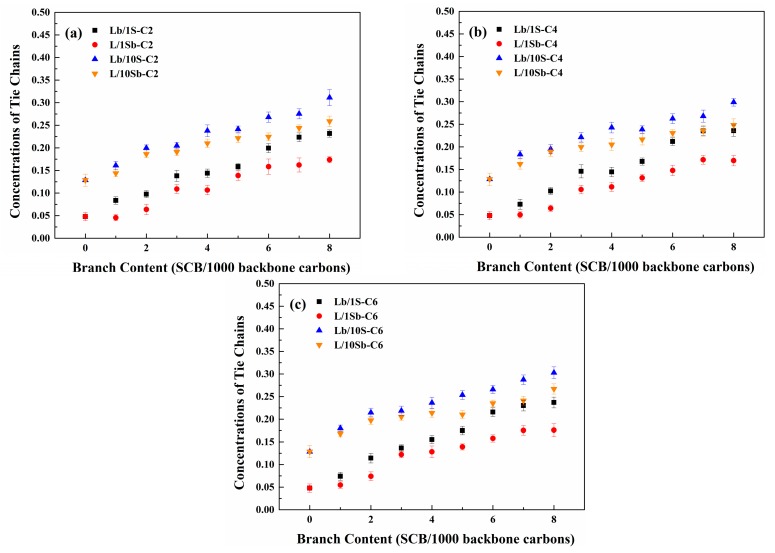
SCBD dependence of tie chains concentration for complex BPE models and simple BPE models with (**a**) ethyl branches, (**b**) butyl branches and (**c**) hexyl branches. Reprinted the data for the simple BPE models with permission from Ref. [27]. Copyright 2018 Elsevier Ltd.

Next, we turn our attention to reveal the SCB effect on the tie chain formation process of BPE models. Figure 11 shows the SCBD effect on the tie chains concentration for the complex BPE models and simple BPE models with different branch length. The tie chains concentration is defined as the ratio of atoms in the tie chains to the total carbons of the system [27]. Comparing the Lb/10S system and the Lb/1S system, the tie chains concentration of the Lb/10S system is higher than that of the Lb/1S system. The differences in the tie chains concentration between these two systems decreased with the increasing branch content. This may also be attributed to the lower MSL of the Lb/10S system as compared to that of the Lb/1S system, and the MSL differences between these two systems decreased with the increasing branch content, as shown in Table S1 in the supplementary material. Therefore, the tie chains concentration depends on the MSL of the system. The tie chains concentration increased with the decrease of MSL of the system. However, the tie chains concentration of the L/10Sb system is higher than that of the L/1Sb system despite the fact that the MSL of the L/10Sb system is longer than that of L/1Sb system. This may be also due to the more nuclei formed in the L/10Sb system when compared with that formed in the L/1Sb system, as shown in Figure S4 in the supplementary material, so more tie chains will form in the L/10Sb system.

The tie chains concentration increased with the increasing branch content both for the two kinds of complex BPE models. This may be due to the decreased crystalline and amorphous thicknesses as branch content increased, and so that more tie chains will form [27,61]. When branch content increased to about 6 SCB/1000C, the increase of the tie chains concentration became slow. This observation is in agreement with the result of simple BPE models [27]. Huang and Brown also found such a critical branch content for tie chain fraction at about 6 SCB/1000C [62]. According to the simulation work of Moyassari et al., this critical branch content is about 2.8 SCB/1000C [61].

Comparing the two types of complex BPE models, it is clearly observed that the tie chains concentration of Lb/10S system is higher than that of L/10Sb system. For simple BPE models, the same phenomenon was observed [27]. Krishnaswamy et al. inferred that blends with short-chain branching (SCB) incorporated in the longer chains show outstanding mechanical properties which may result from the higher tie chains concentration of these blends [48]. Therefore, BPE models with SCBs incorporated in the long chain can form more tie chains in comparison with the case of SCBs incorporated in the short chain for all the BPE models. Furthermore, it is intriguing to find that the differences in the tie chains concentrations between Lb/10S systems and L/10Sb systems are much lower than those between Lb/1S systems and L/1Sb systems. This may also result from the comparable MSL between the two types of complex BPE models, but very different MSL between the two types of simple BPE models. These results seem to accord well with the results of crystallinity for the BPE models. Hence, the SCBD effect on the tie chains concentration of the complex BPE models was much weaker than on that of the simple BPE models.

**Figure 12:**
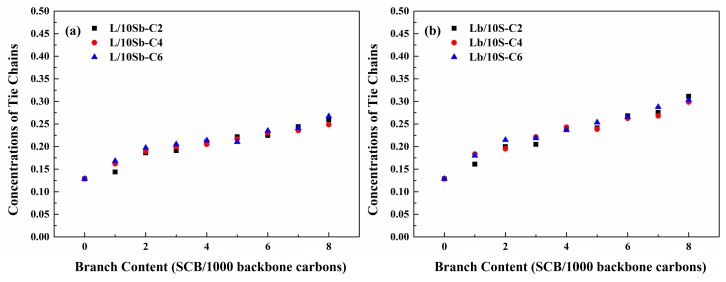
Branch length dependence of tie chains concentration for (**a**) L/10Sb systems and (**b**) Lb/10S systems.

The branch length dependence of tie chains concentration for the two kinds of complex BPE models is depicted in Figure 12. As observed, for all the two kinds of complex BPE models, the tie chains concentrations are insensitive to branch length. This behavior is also consistent with that of the crystallinity of these models. For the simple BPE models, the tie chains concentration was not influenced by branch length, too [27]. The main explanation is that crystalline and amorphous thicknesses are independent of branch length when branch length is smaller than 10 CH_2_ [25]. As a result, the tie chains concentrations for both complex BPE models and simple BPE models are insensitive to branch length.

## 4. Conclusions

Molecular dynamics simulation was conducted to study the molecular mechanism of SCB, especially the MSL and SCBD effect on the BPE crystallization process. The induction time of precursor formation and the initial stage of nucleation depend on the MSL of the system. It was found that 60 CH_2_ is the critical MSL for a branched chain to nucleate. SCB incorporated in the long chain leads to a delay of induction period of precursor formation and the nucleation process when compared with that of SCB incorporated in the short chain. Additionally, these processes are further delayed when branch length increases. The crystallinity of BPE models decreased with the decrease of MSL of the systems. The crystallinity of BPE models with SCB incorporated in the long chain are lower than those of BPE models with SCB incorporated in the short chain when branch content exceeds 5 SCB/1000C. This critical branch content was insensitive to branch length. The location of short chain to the long chain is determined by the MSL of the short chain. The tie chains concentration increased with the decrease of MSL of the system. When SCB incorporated in the long chain, the tie chains concentrations of the models are higher than those of the models with SCB incorporated in the short chain. The tie chain concentrations of the models are not influenced by branch length. The SCBD effect on crystallinity and the tie chains concentrations of the complex BPE models were much weaker than on that of simple BPE models. This simulation work clearly revealed the molecular mechanism of SCB, especially the effects of MSL and SCBD on the initial stage of nucleation, crystallization process and the tie chain formation process of the complex BPE models, which are similar to the normal industrial BPEs as the weight ratio of the long PE chain to the short PE chain in the BPE models is about 1:1. Further investigations of SCB effects on BPE models with another bimodal MWD are still in progress.

## Figures and Tables

**Table 1:** Characteristics of the branched complex BPE models with different branch content and SCBD ^a^.

Complex BPE Model	Branch	Branch Content (SCB/1000 Backbone Carbons)	Methylene Sequence Length (CH_2_)	Total Number of CH_2_
HDPE	None	0	-	20,000
Lb/10S-C2-1	Ethyl	1	476	20,036
L/10Sb-C2-1	Ethyl	1	334	20,060
Lb/10S-C2-2	Ethyl	2	244	20,084
L/10Sb-C2-2	Ethyl	2	200	20,080
Lb/10S-C2-3	Ethyl	3	164	20,124
L/10Sb-C2-3	Ethyl	3	142	20,060
Lb/10S-C2-4	Ethyl	4	124	20,204
L/10Sb-C2-4	Ethyl	4	112	20,240
Lb/10S-C2-5	Ethyl	5	100	20,300
L/10Sb-C2-5	Ethyl	5	92	20,320
Lb/10S-C2-6	Ethyl	6	82	20,162
L/10Sb-C2-6	Ethyl	6	76	20,120
Lb/10S-C2-7	Ethyl	7	70	20,150
L/10Sb-C2-7	Ethyl	7	66	20,180
Lb/10S-C2-8	Ethyl	8	62	20,302
L/10Sb-C2-8	Ethyl	8	58	20,180

^a^ The design of branched complex BPE models with butyl or hexyl branches (not shown here) are the same as the complex BPE models with ethyl branches, and the differences are the branch length.

## Supplementary Materials

The following are available online at https://www.mdpi.com/2073-4360/11/11/1840/s1, Figure S1: Box size dependence of crystallinity (X_c_) for HDPE model in Table 1 in the manuscript. *l*_e_* is the normalized distance from the end of the polymer chain to the closest edge of the box.; Figure S2. Branch length dependence of induction time (*t*_p_*) for (a) L/1Sb systems and; Figure S3. Crystallization curves of different branch content for (a) L/10Sb-C2, (b) Lb/10S-C2, (c) L/10Sb-C4, (d) Lb/10S-C4, (e) L/10Sb-C6, (f) Lb/10S-C6 systems; Figure S4. Number of nuclei of the ethyl branched complex BPE model chains and simple BPE model chains at the end of the simulation; Figure S5. Branch length dependence of SOP and X_c_ for (a) L/10Sb systems and (b) Lb/10S systems; Table S1: Methylene sequence length (MSL) of the complex BPE model chains and the simple BPE model chains.

## Abbreviations

SCB	Short-chain branching
MSL	Methylene sequence length
SCBD	Short-chain branching distribution
BPE	Bimodal polyethylene
PE	Polyethylene
MWD	Molecular weight distribution
MD	Molecular dynamics
ADMET	Acyclic diene metathesis
HDPE	High-density polyethylene
SOP	Site order parameter
Xc	Crystallinity
ICF	Interchain contact fraction
t_p_*	Induction time
LCP	Long-chain proportion
t_n_*	Formation time of the first stable nucleus
