# Supplementary material for: Dominant Effects of Short-Chain Branching on the Initial Stage of Nucleation and Formation of Tie Chains for Bimodal Polyethylene as Revealed by Molecular Dynamics Simulation
Source: Polymers (Basel). 2019 Nov 8;11(11):1840. doi: 10.3390/polym11111840 (PMC6918436; doi:10.3390/polym11111840)
Supplement: Supplementary file 1 [file polymers-11-01840-s001.pdf]

Supplementary Material for: Dominant Effects of Short-Chain Branching on the Initial Stage of Nucleation and Formation of Tie Chains for Bimodal Polyethylene as Revealed by Molecular Dynamics Simulation

Yanling Hu [1], Yunqi Shao [1], Zhen Liu [1], Xuelian He [1,*] and Boping Liu [2,*]

[1] Shanghai Key Laboratory of Multiphase Material Chemical Engineering, East China University of Science and Technology, Shanghai 200237, China; huyl@mail.ecust.edu.cn (Y.H.); yunqi_shao@mail.ecust.edu.cn (Y.S.); liuzhen@ecust.edu.cn (Z.L.)

[2] College of Materials and Energy, South China Agricultural University, Guangzhou 510642, China; boping@scau.edu.cn

* Correspondence: hexl@ecust.edu.cn (X.H.); boping@scau.edu.cn (B.L.)

Received: 9 October 2019; Accepted: 5 November 2019; Published: date

1. Effect of the Box Size on the Crystallinity of Complex BPE Model

Fig. S1 shows the box size dependence of crystallinity (Xc) for HDPE model in Table 1 in the manuscript. l_e* is the normalized distance from the end of the polymer chain to the closest edge of the box. As observed, the value of Xc for HDPE model increased with the increase of the normalized distance l_e*, up to about 6σ. Above 6σ, the value of Xc for HDPE model was almost stable at around 0.45. So in this paper, we chose the value of l_e* as 25σ to eliminate the effect of box size on the crystallinity of all the models. Note that the side length of the box was much larger than the normalized distance l_e*(when l_e* was set at about 25σ, the corresponding side length of the box was about 1100σ).

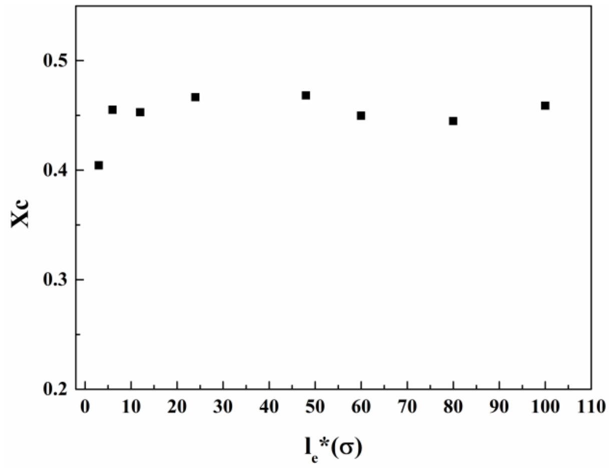

Figure S1. Box size dependence of crystallinity (Xc) for HDPE model in Table 1 in the manuscript. l_e* is the normalized distance from the end of the polymer chain to the closest edge of the box.

2. Comparison between Simulation Temperature and Real Experimental Temperature

In order to make a rough comparison with the real experimental melting temperature of BPE, we calculated the melting temperature of HDPE model (non-branched complex BPE model) in Table 1. The final semicrystalline state (state of the end of the simulation) of this model was then melted by heating it from T* = 3.0 – 8.0 at a same heating rate $\Gamma^* = 2.5 \times 10^{-4}\ \tau^{-1}$, and the melting temperature of this model was T^*_m = 7.5. The corresponding experimental melting temperatures for BPE are in a range of 132 - 137 °C [1]. High simulation temperature T* = 8.0 was corresponding to the real experimental temperature which was higher than the melting temperature of BPE.

3. References

1. Krishnaswamy, R.K.; Yang, Q.; Fernandez-Ballester, L.; Kornfield, J.A.

Effect of the distribution of short-chain branches on crystallization kinetics and mechanical properties of high-density polyethylene. *Macromolecules* **2008**, *41*, 1693-1704.

Other Figures and Tables

Table S1. Methylene sequence length (MSL) of the complex BPE model chains and the simple BPE model chains

Branch Content (SCB/1000 backbone carbons)	Methylene Sequence Length (MSL)/CH_2			
	**Lb/10S**	**L/10Sb**	**Lb/1S**	**L/1Sb**
1	476	334	1000	100
2	244	200	500	50
3	164	142	332	34
4	124	112	250	26
5	100	92	200	20
6	82	76	166	16
7	70	66	142	14
8	62	58	124	12

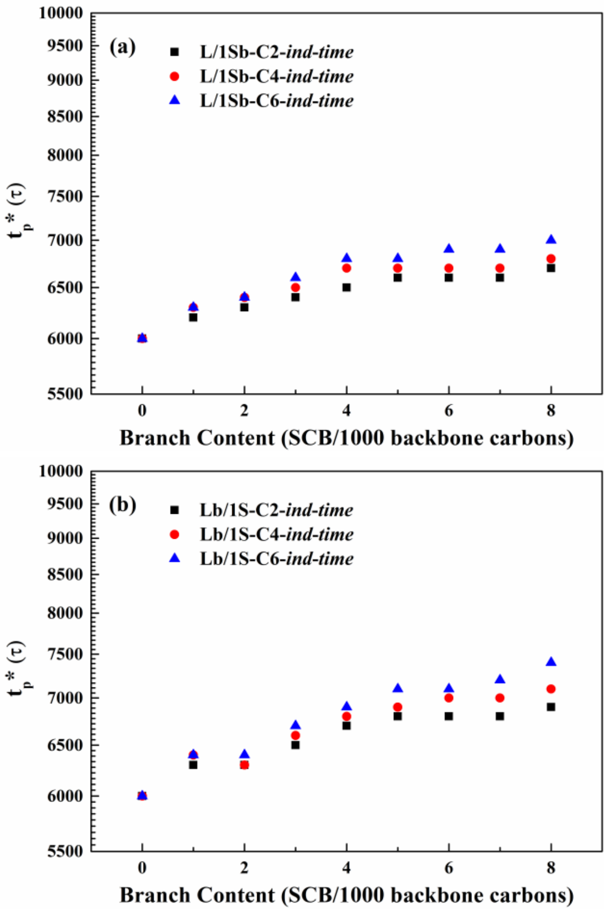

Figure S2. Branch length dependence of induction time (t_p*) for (a) L/1Sb systems and (b) Lb/1S systems.

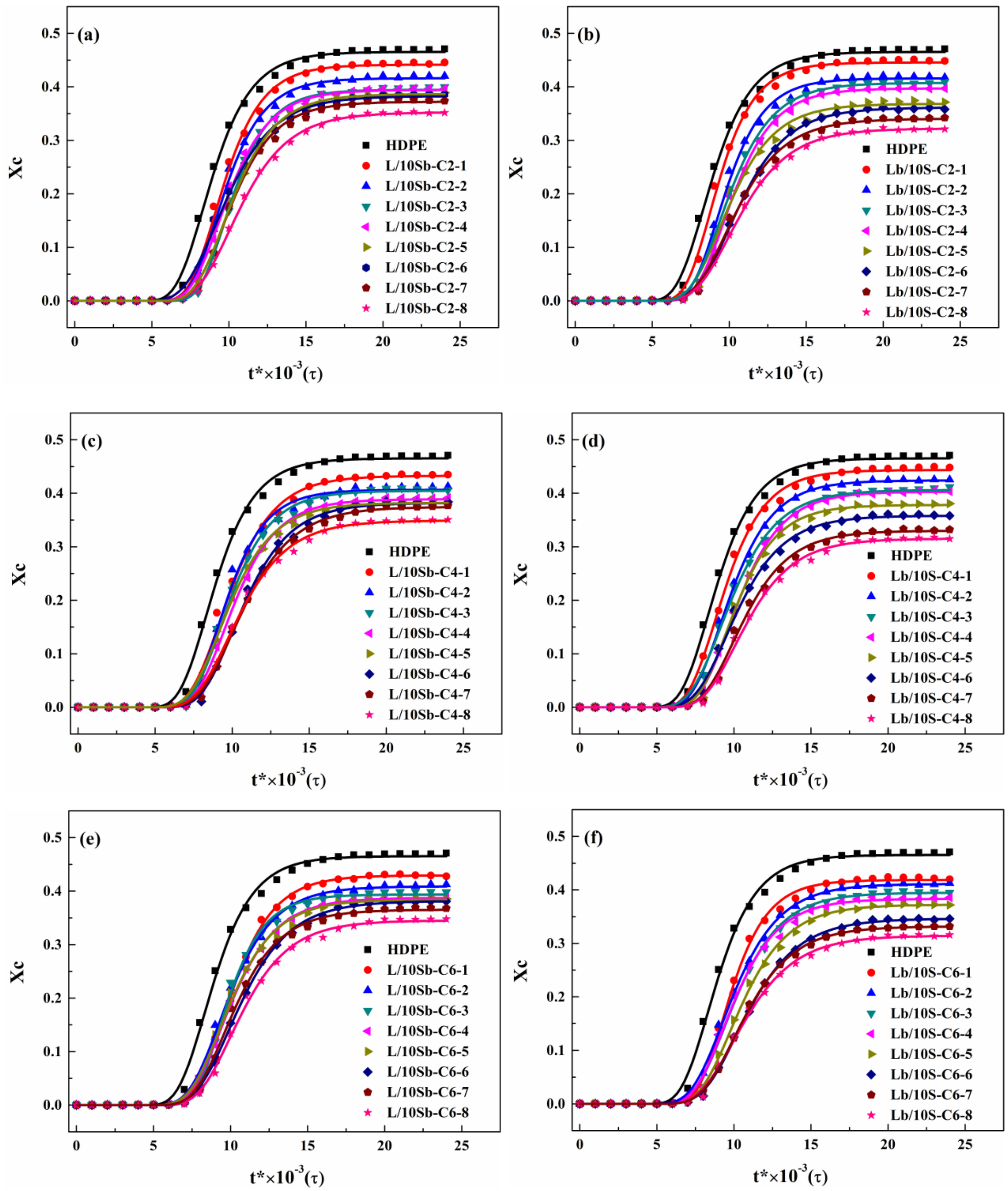

Figure S3. Crystallization curves of different branch content for (a) L/10Sb-C2, (b) Lb/10S-C2, (c) L/10Sb-C4, (d) Lb/10S-C4, (e) L/10Sb-C6, (f) Lb/10S-C6 systems.

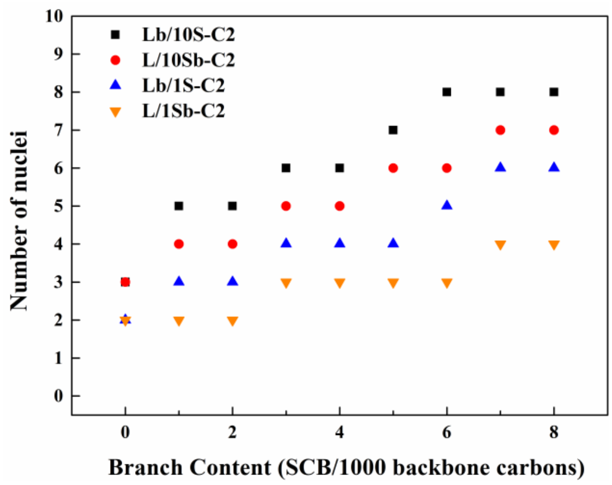

Figure S4. Number of nuclei of the ethyl branched complex BPE model chains and simple BPE model chains at the end of the simulation.

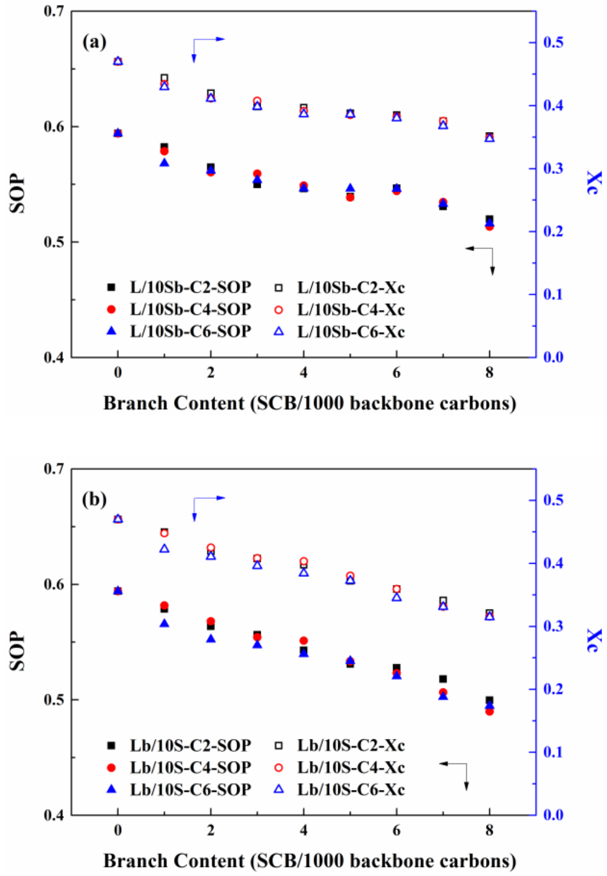

Figure S5. Branch length dependence of SOP and Xc for (a) L/10Sb systems and (b) Lb/10S systems.
